# Efficient VLSI Architecture for Training Radial Basis Function Networks

**DOI:** 10.3390/sl30303848

**Published:** 2013-03-19

**Authors:** Zhe-Cheng Fan, Wen-Jyi Hwang

**Affiliations:** Department of Computer Science and Information Engineering, National Taiwan Normal University, Taipei 116, Taiwan; E-Mail: a205152@hotmail.com

**Keywords:** reconfigurable computing, system on programmable chip, FPGA, radial basis function, fuzzy *C*-means

## Abstract

This paper presents a novel VLSI architecture for the training of radial basis function (RBF) networks. The architecture contains the circuits for fuzzy *C*-means (FCM) and the recursive Least Mean Square (LMS) operations. The FCM circuit is designed for the training of centers in the hidden layer of the RBF network. The recursive LMS circuit is adopted for the training of connecting weights in the output layer. The architecture is implemented by the field programmable gate array (FPGA). It is used as a hardware accelerator in a system on programmable chip (SOPC) for real-time training and classification. Experimental results reveal that the proposed RBF architecture is an effective alternative for applications where fast and efficient RBF training is desired.

## 1. Introduction

Radial basis function (RBF) [1,2] networks have been found to be effective for many real world applications due to their ability to approximate complex nonlinear mappings with a simple topological structure. A basic RBF network consists of three layers: An input layer, a hidden layer with a nonlinear kernel, and a linear output layer. The Gaussian function is commonly used for the nonlinear kernel.

The parameter estimation of RBF networks concerns the optimization of centers of the Gaussian kernels as well as the connecting weights between neurons. The estimation of the above parameters is carried out using two-staged learning strategies. In the first stage, cluster analysis is implemented to calculate the appropriate values of the centers. In the second stage, supervised optimization procedures are involved in the optimal estimation of the connecting weights.

One effective clustering approach for finding centers is the *K*-means algorithm [2]. However, because of iterative crisp clustering operations, the results of the *K*-means algorithm are sensitive to the selection of initial centers. In addition, the computation complexities of the algorithm are high for large set of training vectors. The fuzzy *C*-means (FCM) algorithm and its variants [3,4] are the effective alternatives for finding the centers. The FCM adopts a fuzzy partitioning approach for clustering. It allows the training vectors to belong to several clusters simultaneously, with different degrees of membership. Although the FCM is also an iterative algorithm, the clustering performance is less susceptible to the initial centers. However, the fuzzy clustering involves the computation of degree of membership, which may be very computationally expensive as the number of training vectors and/or the number of clusters become large. The particle swarm optimization (PSO) techniques [5,6] are also beneficial for computing the centers. The techniques can operate in conjunction with fuzzy clustering [6] for attaining near optimal performance. Nevertheless, when the number of particles and/or the dimension associated with each particle are large, the real-time RBF training may still be difficult.

To estimate the connecting weights in the output layer, least mean square (LMS) methods are the commonly used techniques. However, basic LMS approach involves the computation of the inverse of the correlation matrix in the hidden layer of the RBF network. When the size of the hidden layer and/or training set becomes large, the inverse matrix computation may become a demanding task. The requirement of inverse matrix operations can be lifted by the adoption of recursive LMS. Nevertheless, because extensive matrix multiplications are required, especially for large hidden layer and/or training set, the recursive LMS still has high computational complexities.

Many efforts have been made to expedite RBF training. The techniques in [7–9] focus on reducing the training time for centers. The algorithm presented in [7] uses subtractive clustering. The fast technique in [8] modifies the basic *K*-means algorithm. The center updating in [9] is based on an incremental scheme. In [10], an incremental technique is used for the updating of connecting weights in the output layer. These fast algorithms are implemented by software. Therefore, only moderate acceleration can be achieved. Moreover, for the incremental algorithms [9,10], inappropriate selection of learning rate may severely degrade the training performance.

The algorithm in [11] is suited for finding centers by hardware. It involves only replicating selected training vectors as centers. The number of centers produced by the algorithm can be controlled by the radius parameter [11]. Nevertheless, the mapping from the radius parameter to the number of centers may vary for different training sets. It may then be difficult to find a search scheme efficient for seeking optimal radius parameters subject to a constraint on the RBF network hidden layer size for different training sets.

The analog hardware implementation [12,13] for RBF training has been found to be effective for reducing the computation time. However, these architectures are difficult to be directly used for digital devices. Digital hardware realization of RBF in [14] focuses only on the implementation of topological structure of the networks. The training of the centers in the hidden layer and the connecting weights in output layer are performed by software. Other RBF-based applications in embedded systems [15,16] are also implemented in a similar fashion.

In [17,18], the digital hardware architectures for RBF training have been presented. However, the training for centers is not considered in [17]. The training for connecting weights is based on incremental operations. The architecture in [18] is able to train both the centers and the connecting weights. All training operations are performed incrementally. Although the incremental training is more suitable for hardware implementation, the performance is dependent on the selection of learning rate. The value of learning rate may be truncated for the finite precision hardware implementation. Similar to the improper learning rate selection, the truncation of learning rate may result in a poor local optimum for RBF training.

The goal of this paper is to present a novel hardware architecture for real-time RBF training. The architecture is separated into two portions: the FCM circuit, and the recursive LMS circuit. The FCM circuit is designed for the training of centers in the hidden layer. The recursive LMS circuit is adopted for the training of connecting weights in the output layer. Both the FCM and the recursive LMS circuits are digital circuit requiring no learning rate.

The FCM circuit features low memory consumption and high speed computation. In the circuit, the usual iterative operations for updating the membership matrix and cluster centers are merged into one single updating process to evade the large storage requirement. In addition, the single updating process is implemented by a novel pipeline architecture for enhancing the throughput of the FCM training. In our design, the updating process is divided into three steps: Pre-computation, membership coefficients updating, and center updating. The pre-computing step is used to compute and store information common to the updating of different membership coefficients. This step is beneficial for reducing the computational complexity for the updating of membership coefficients. The membership updating step computes new membership coefficients based on a fixed set of centers and the results of the pre-computation step. The center updating step computes the center of clusters using the current results obtained from the membership updating step. The final results of this step will be used for subsequent RBF processing.

The recursive LMS circuit performs weight updating using the centers obtained from the FCM circuit. The recursive LMS algorithm involves large number of matrix operations. To enhance the computational speed of matrix operations, an efficient block computation circuit is proposed for parallel multiplications and additions. The block dimension is identical to the number of nodes in the hidden layer so that all the connecting weights can be updated concurrently. To facilitate the block computation, buffers for storing intermediate results of recursive LMS algorithm are implemented as shift registers allowing both horizontal and vertical shifts. Columns and rows of a matrix can then easily be accessed. All matrix operations share the same block computation circuit for lowering area cost. Therefore, the proposed block computation circuit has the advantages of both high speed computation and low area cost for recursive LMS.

To demonstrate the effectiveness of the proposed architecture, a hardware classification system on a system-on-programmable-chip (SOPC) platform is constructed. The SOPC system may be used as a portable sensor for real-time training and classification. The system consists of the proposed architecture, a softcore NIOS II processor [19], a DMA controller, and a SDRAM. The proposed architecture is adopted for online RBF training with the training vectors stored in the SDRAM. The DMA controller is used for the DMA delivery of the training vectors. The softcore processor is used for coordinating the SOPC system. Some parameters of the RBF training process are not fixed by hardware. They can be modified by the softcore processor to enhance the flexibility of the SOPC system. As compared with its software counterpart running on Intel I5 CPU, our system has significantly lower computational time for large training set. All these facts demonstrate the effectiveness of the proposed architecture.

## 2. The RBF Networks

**Figure 1.**
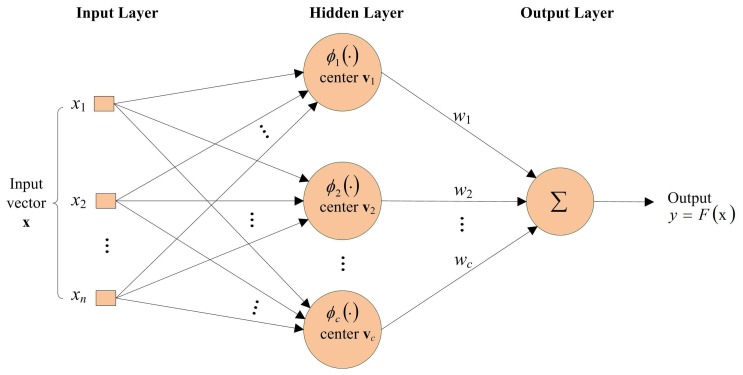
A typical RBF network.

This section reviews some basic facts of RBF networks. A typical RBF network revealed in Figure 1 consists of an input layer, a hidden layer and an output layer. The input layer contains *n* source nodes, where *n* is the dimension of the input vector x. The hidden layer consists of *c* neurons. A kernel function is associated with each neuron. A typical kernel function used in the RBF networks is the Gaussian kernel. Let *ϕ_i_* be the Gaussian kernel associated with the *i*-th neuron, which is defined as

(1) $$\phi_{i}(x)=\exp(-\frac{1}{2\sigma^{2}}{\left\|x-v_{i}\right\|}^{2})$$

The v*_i_* in Equation (1) is the center associated with the *i*-th neuron. Both x and v*_i_* have the same dimension *n*. The *σ*^2^ in Equation (1) is termed the radius of the Gaussian kernel. It is assumed in this study that all kernels have the same radius.

The output layer contains only one neuron. Let *ŷ* be the output of the neuron, which is given by

(2) $$\hat{y}=\sum_{i=1}^{c}\omega_{i}\phi_{i}(x)$$

The *w_i_* is termed the connecting weights between the *i*-th neuron in the hidden layer and the output neuron. The RBF training usually involves the training of centers v*_i_* and connecting weights *w_i_i* = l,…,*c*.

### 2.1. FCM for the Training of Centers

The FCM can be effectively used for the training of centers. Let *X* = {x_1;_…, x*_t_*} be a set of training vectors for RBF training, where *t* is the number of training vectors. The FCM computes v*_i_, i* = 1,…, *c*. by separating *X* into *c* clusters. The v*_i_* is then the center of cluster *i*. The FCM involves minimization of the following cost function:

(3) $$J=\sum_{i=1}^{c}\sum_{k=1}^{t}u_{i,k}^{m}{\left\|x_{k}-v_{i}\right\|}^{2}$$

where *u_i,k_* is the membership of *x_k_* in class *i*, and *m* > 1 indicates the degree of fuzziness. The cost function *J* is minimized by a two-step iteration in the FCM. In the first step, the centers v_1;_…, v*_c_*, are fixed, and the optimal membership matrix {*u_i,k_, i* = 1,…, *c*, *k* = 1,…, *t*} is computed by

(4) $$u_{i,k}={\left(\sum_{j=1}^{c}{\left(\left\|x_{k}-v_{i}\right\|/\left\|x_{k}-v_{j}\right\|\right)}^{2/\left(m-1\right)}\right)}^{-1}$$

After the first step, the membership matrix is then fixed, and the new center *v_i_* is obtained by

(5) $$v_{i}=\left(\sum_{k=1}^{c}u_{i,k}^{m}x_{k}\right)/\left(\sum_{k=1}^{t}u_{i,k}^{m}\right)$$

The iteration continues until the convergence of *J*. From Equations (3) and (5), it follows that the membership matrix needs to be stored for the computation of cost function and centers. As the size of the membership matrix grows with the product of *t* and *c*, the storage size required for the FCM may be impractically large for hardware implementation.

### 2.2. Recursive LMS for the Training of Connecting Weights

The training of connecting weights is also based on the training set *X* = {x_1_,…, x*_t_*}. Let *ŷ_k_* be the output of RBF network when the input is the *k*-th training vector x*_k_* ϵ *X*. That is, from Equation (2)

$${\hat{y}}_{k}=\sum_{i=1}^{c}\omega_{i}\phi_{i}\left(x_{k}\right)$$

Define

(6) $$a_{k}={\left[\begin{array}{cccc} \phi_{1}(x_{k}) & \phi_{2}(x_{k}) & \ldots & \phi_{c}(x_{k}) \end{array}\right]}^{T}$$

(7) $$w={\left[\begin{array}{cccc} \omega_{1} & \omega_{2} & \ldots & \omega_{c} \end{array}\right]}^{T}$$

It then follows that

(8) $${\hat{y}}_{k}=a_{k}^{T}w$$

In addition, let

(9) $$\hat{y}={\left[\begin{array}{cccc} {\hat{y}}_{1} & {\hat{y}}_{2} & \ldots & {\hat{y}}_{t} \end{array}\right]}^{T}$$

be the vector containing all the outputs for the training set *X*, and

(10) $$A=\left[\begin{array}{c} a_{1}^{T}\\ a_{2}^{T}\\ \ldots \\ a_{t}^{T} \end{array}\right]$$

From Equations (8) and (10), we see that

(11) $$\hat{y}=\text{Aw}$$

Define

(12) $$y={\left[\begin{array}{cccc} y_{1} & y_{2} & \ldots & y_{t} \end{array}\right]}^{T}$$

as the vector consisting of all the *desired* outputs for the training set *X*, where *y_k_* is the *desired* output associated with the input x*_k_*. Let

(13) $$E=\sum_{k=1}^{t}{\left({\hat{y}}_{k}-y_{k}\right)}^{2}$$

be the square distance between y and ŷ. It can be shown that [2] the LMS estimate of w minimizing *E* is given by

(14) $$w={\left(A^{T}A\right)}^{-1}A^{T}y$$

Finding w based on Equation (14) involves the operations of matrix inverse and multiplication. The LMS estimate of w may therefore be difficult to be implemented by hardware when number of training vectors *t* and/or the number of centers *c* are large. An effective alternative to the LMS method is the recursive LMS. Given training set *X*, instead of computing w in one shot using Equation (14), the recursive LMS computes w incrementally.

Suppose training vectors become available in sequential order. Without loss of generality, assume x_1;_…, x*_k_*_−1_ and the corresponding outputs *y*_1_,…, *y_k_*_−1_ are available. Define

(15) $$y_{k-1}={\left[\begin{array}{cccc} y_{1} & y_{2} & \ldots & y_{k-1} \end{array}\right]}^{T}$$

Based on x_1;_…, x*_k_*_−1_ the first (*k* – 1) rows of A can be evaluated. Let

(16) $$A_{k-1}=\left[\begin{array}{c} a_{1}^{T}\\ a_{2}^{T}\\ \ldots \\ a_{t}^{T} \end{array}\right]$$

be the first (*k* – 1) rows of A. The LMS estimate of w based on A*_k_*_−1_ and y*_k_*_−1_, denoted by w*_k_*_−1_, can be computed by Equation (14) as

$$w_{k-1}={\left(A_{k-1}^{T}A_{k-1}\right)}^{-1}A_{k-1}^{T}y_{k-1}$$

Suppose a new data pair (x*_k_*, *y_k_*) becomes available. Then instead of using all the *k* available data pairs to recompute the w*_k_*, the recursive LMS takes the advantage of the w*_k_*_−1_ already available to obtain w*_k_*. Define

(17) $$P_{k-1}={\left(A_{k-1}^{T}A_{k-1}\right)}^{-1}$$

It can then be shown that

(18) $$P_{k}=P_{k-1}-\frac{P_{k-1}a_{k}a_{k}^{T}P_{k-1}}{1+a_{k}^{T}P_{k-1}a_{k}}$$

and

(19) $$w_{k}=w_{k-1}+P_{k}a_{k}(y_{k}-a_{k}^{T}w_{k-1)}$$

To initialize the algorithm, set

(20) $$P_{0}=\lambda^{-1}I$$

(21) $$w_{0}=0$$

where λ is a small positive number.

## 3. The Architecture

**Figure 2.**
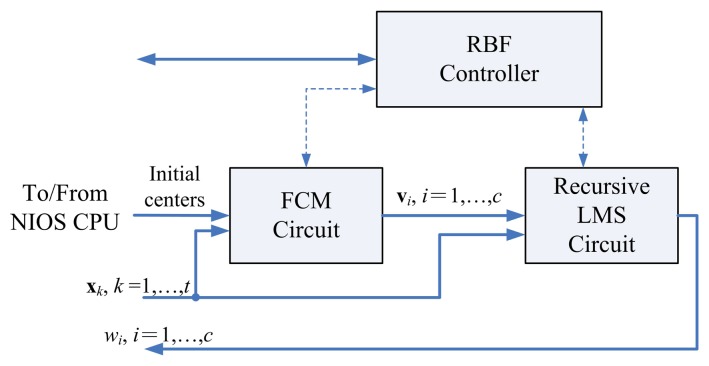
The proposed RBF architecture.

As shown in Figure 2, the proposed architecture for RBF training can be separated into two units: the FCM unit and the recursive LMS unit. The goal of the FCM unit is to compute the centers v*_i_*, *i* = 1,…, *c*, given the training set *X*. Based on the centers produced by FCM unit, and the training set *X*, the recursive LMS unit finds the weights *w_i_,i* = 1,…, *c*.

## 4. FCM Unit

**Figure 3.**
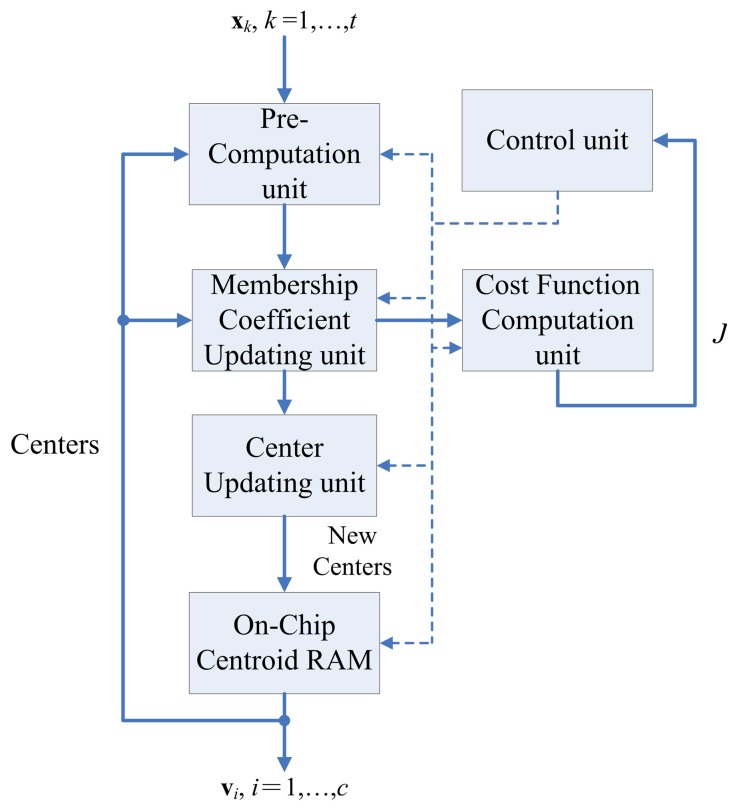
The FCM architecture.

Figure 3 shows the architecture of the FCM unit, which contains six sub-units: The pre-computation unit, the membership coefficients updating unit, center updating unit, cost function computation unit, FCM memory unit, and control unit. The operations of each sub-unit are stated below.

### 4.1. Pre-Computation Unit

The pre-computation unit is used for reducing the computational complexity of the membership coefficients calculation. Observe that *u_i,k_* in Equation (4) can be rewritten as

(22) $$u_{i,k}={\left\|x_{k}-v_{i}\right\|}^{-2/\left(m-1\right)}R_{k}^{-1}$$

where

(23) $$R_{k}=\sum_{j=1}^{c}{\left(1/{\left\|x_{k}-v_{j}\right\|}^{2}\right)}^{1/\left(m-1\right)}$$

Given x*_k_* and centers v_1_,…, v*_c_*, membership coefficients *u*_1_*_,k_*,…*u_c,k_* have the same *R_k_*. Therefore, the complexity for computing membership coefficients can be reduced by calculating *R_k_* in the pre-computation unit. For the sake of simplicity, we set *m* = 2 for our design. Consequently, *R_k_* can be viewed as the sum of 1/‖x*_k_* − v*_j_*‖^2^.

**Figure 4.**
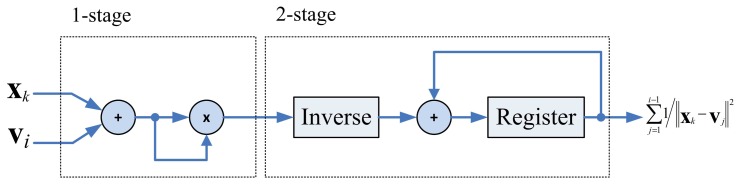
The architecture of pre-computation unit.

The architecture for computing *R_k_* is depicted in Figure 4, which can be divided into two stages. The first stage evaluates ‖x*_k_* − v*_j_*‖^2^. The second stage first finds the inverse of ‖x*_k_* − v*_j_*‖^2^, and then accumulate this value with $\sum_{j=1}^{i-1}1/{\left\|x_{k}-v_{j}\right\|}^{2}$.

### 4.2. The Membership Updating Unit

**Figure 5.**
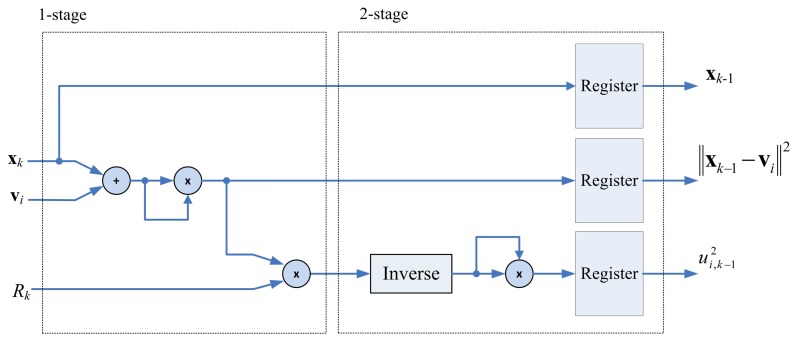
The architecture of membership coefficients computation unit.

Based on Equation (22), the membership updating unit uses the computation results of the pre-computation unit for calculating the membership coefficients. Figure 5 shows the architecture of the membership coefficients updating unit. It can be observed from Figure 5 that, given a training data x*_k_*, the membership coefficients computation unit computes $u_{i,k}^{2}$for *i* = 1,…, *c*, one at a time. The circuit can be separated to two stages. The first stage and the second stage of the pipeline are used for computing ‖x*_k_* − v*_i_*‖^2^*R_k_* and $u_{i,k}^{2}$respectively.

### 4.3. Center Updating Unit

The center updating unit incrementally computes the center of each cluster. The major advantage for the incremental computation is that it is not necessary to store the entire membership coefficients matrix for the center computation. Define the incremental center for the *i*-th cluster up to data point x*_k_* as

(24) $$vi(k)=\left(\sum_{n=1}^{k}u_{i,n}^{m}x_{n}\right)/\left(\sum_{n=1}^{k}u_{i,n}^{m}\right)$$

when *k* = *t*, v*_i_*(*k*) then is identical to the actual center v*_i_* given in Equation (5).

**Figure 6.**
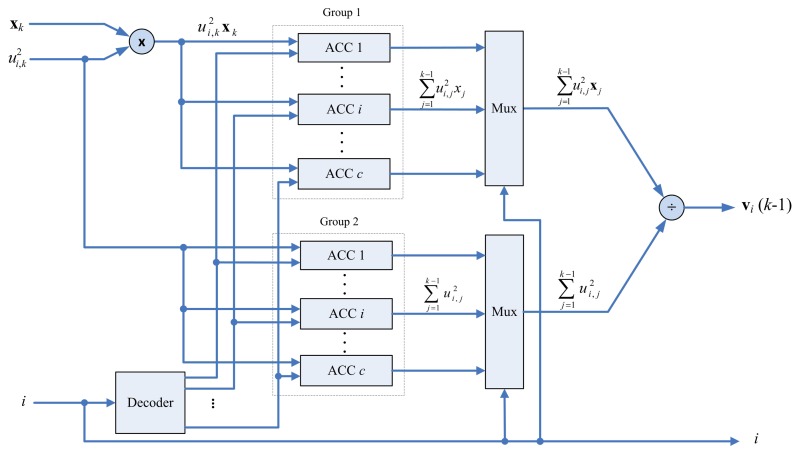
The architecture of center updating unit.

The architecture of the center updating unit is depicted in Figure 6. It contains a multiplier, an accumulator (ACC) array and a divider. There are two groups in the ACC array. The *i*-th ACC in the first group contains the accumulated sum $\sum_{j=1}^{k-1}x_{j}\mu_{i,j}^{2}$. Moreover, the *i*-th ACC in the second group contains the accumulated sum $\sum_{j=1}^{k-1}\mu_{i,j}^{2}$. The outputs of the array are used for computing v*_i_*(*k*) using a divider.

### 4.4. Cost Function Computation Unit

Similar to the center updating unit, the cost function unit incrementally computes the cost function *J*. Define the incremental cost function *J*(*i*, *k*) as

(25) $$J(i,k)=\sum_{z=1}^{k}\sum_{j=1}^{i}u_{j,z}^{2}{\left\|x_{z}-v_{j}\right\|}^{2}$$

**Figure 7.**
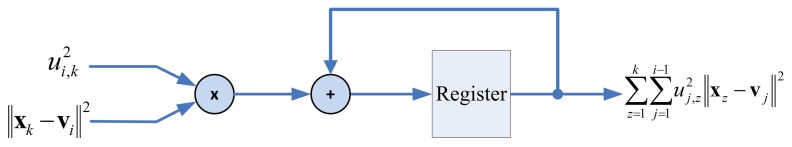
The architecture of cost function computation unit.

As shown in Figure 7, the circuit receives $u_{i,k}^{2}$and ‖x*_k_*−v*_i_*‖^2^ from the membership coefficients updating unit. The product $u_{i,k}^{2}{\left\|x_{k}-v_{i}\right\|}^{2}$is then accumulated for computing *J*(*i, k*) in Equation (25).

When *i* = *c* and *k* = *t, J*(*i, k*) then is identical to the actual cost function *J* given in Equation (3). Therefore, the output of the circuit becomes *J* as the cost function computations for all the training vectors are completed.

### 4.5. FCM Memory Unit

This unit is used for storing the centers for FCM clustering. There are two memory banks (Memory Bank 1 and Memory Bank 2) in the on-chip center memory unit. The Memory Bank 1 stores the current centers v_1_,…, v*_c_*. The Memory Bank 2 contains the new centers v_1_,…, v*_c_* obtained from the center updating unit. Only the centers stored in the Memory Bank 1 are delivered to the pre-computation unit and membership updating unit for the membership coefficients computation. The updated centers obtained from the center updating unit are stored in the Memory Bank 2. Note that the centers in the Memory Bank 2 will not replace the centers in the Memory Bank 1 until all the input training data points x*_k_*, *k* = 1,…, *t*, are processed.

### 4.6. Employment of Shift Registers for Reducing Area Costs for Large Input Vector Dimension n

In the pre-computation unit, membership coefficient updating unit and center updating unit of the FCM, a number of vector operations are required. Each of these operations needs *n* adders, multipliers or dividers to operate in parallel. Therefore, as the input vector dimension *n* becomes large, the area costs will be high.

One way to reduce the area costs is to separate each of the input vectors x*_k_* and centers v*_i_* into *q* segments, where each segment contains only *n/q* elements. The vector operations are then performed over the segments. This requires only *n/q* adders, multipliers or dividers to operate in parallel. To implement the segment-based operations, each of the registers holding the input vectors x*_k_* and centers v*_i_* has to be implemented as a *q*-stage shift register. Each stage of the register consists of *n/q* elements (*i.e.*, one segment). That is, the shift registers are able to fetch or deliver one segment at a time. The shift registers are then connected to an array of *n/q* adders, multipliers or dividers for vector operations with reduced area costs. The vector operations will not be completed until all the segments in the shift registers are processed. Therefore, the latency of the vector operations may increase by *q*-fold.

The shift-register based approach has a number of advantages. First of all, it does not change the basic architectures of the proposed FCM circuit. In fact, the FCM circuits with different *q* values share the same architectures for pre-computation, membership coefficient updating, center updating, and cost function computation. Only the registers holding input training vectors and centers may have different architectures. For the basic FCM circuit with *q* = 1, these registers are the simple *n*-elements parallel-in parallel-out registers. When *q* ≥ 2, these registers become q-stage shift registers with each stage consisting of *n/q* elements.

The second advantage is that it provides higher flexibility to the FCM circuit. It is especially helpful when the input vector dimension *n* is large. In this case, basic design with *q* = 1 is suited only for applications requiring fast speed computation. However, because of the large area costs, it is difficult to implement the circuit in small FPGA devices. This difficulty may be solved by the realization of FCM with larger *q* values, which usually requires significantly lower consumption of hardware resources.

## 5. Recursive LMS Unit

**Figure 8.**
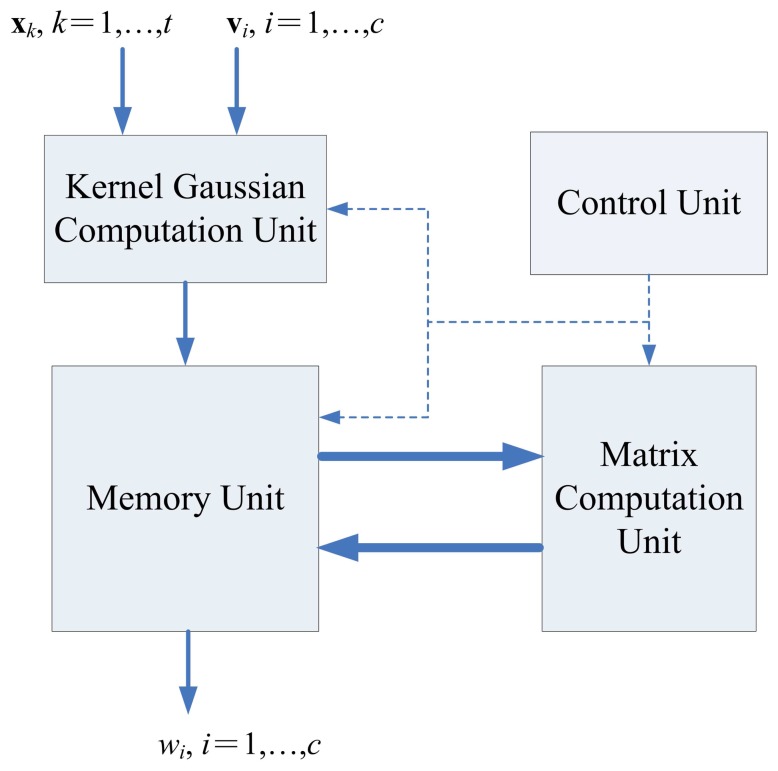
The proposed recursive LMS architecture.

The architecture of recursive LMS unit is shown in Figure 8, which contains kernel Gaussian Computation unit, memory unit and matrix computation unit, and control unit.

### 5.1. Kernel Gaussian Computation Unit

**Figure 9.**
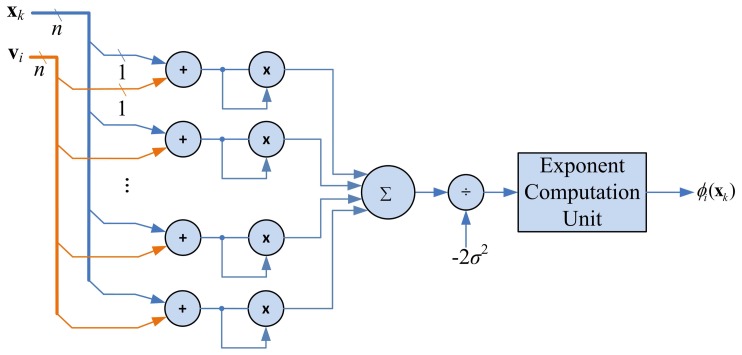
The architecture of kernel Gaussian computation unit.

The goal of kernel Gaussian computation unit is to compute *ϕ_i_*(x) given in Equation (1). Given x*_k_* and the centers v_1_,…, v*_c_*, the kernel Gaussian computation unit calculates the *ϕ*_1_(x_k_),…, *ϕ_c_*(x_k_), sequentially to produce the vector a*_k_*. Figure 9 shows the architecture of the kernel Gaussian computation unit. In addition to adders and multipliers, the architecture contains circuit for computing exponential function. This circuit is implemented by Altera Floating Point Exponent (ALTFP_EXP) Megafunction [20].

Similar to the FCM circuit, the number of adders and multipliers in this unit grows with input vector dimension *n*. When *n* is large, the area costs for implementing the unit will be high. The shift register based approach employed in the FCM circuit can also be used here for reducing the area complexities. In this approach, each of the registers holding x*_k_* and v*_i_* is a *q*-stage shift register. The number of adders and multipliers become *n/q*. The hardware resource consumption can then be lowered.

### 5.2. Memory Unit

**Figure 10.**
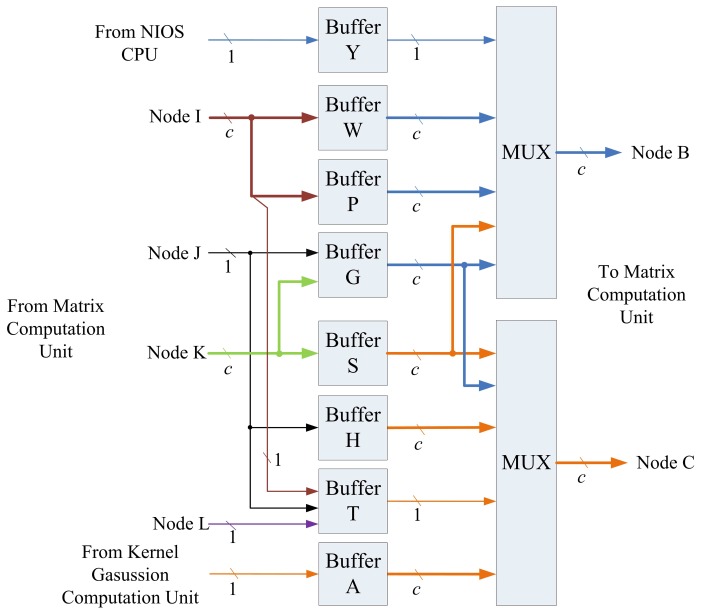
The architecture of memory unit.

The memory unit is used to hold values required for the computation of recursive LMS algorithm shown in Equations (18) and (19). As depicted in Figure 10, there are 8 buffers (Buffers Y, W, P, G, S, H, T, and A) in the memory unit. When x*_k_* is the current training vector, the Buffer A stores a*_k_* obtained from kernel Gaussian computation unit. The Buffer Y contains the *y_k_*. The Buffers P and W consists of P*_k_*_−1_ and w*_k_*_−1_, which are the computation results for the previous training vector x*_k_*_−1_. Based on a*_k_*, *y_k_*, P*_k_*_−1_ and w*_k_*_−1_, the matrix computation unit is then activated for the computation of P*_k_* and w*_k_*. The intermediate results during the computation are stored in the Buffers G, S, H and T. The P*_k_* and w*_k_* are then stored in Buffers P and W for the subsequent operations for the next training vector x*_k_*_+1_. These buffers can operate as parallel-in parallel-out (PIPO), parallel-in serial-out (PISO), serial-in parallel-out (SIPO), and/or serial-in serial-out (SISO) registers. The attributes of these buffers are summarized in Table 1.

### 5.3. Matrix Computation Unit

**Figure 11.**
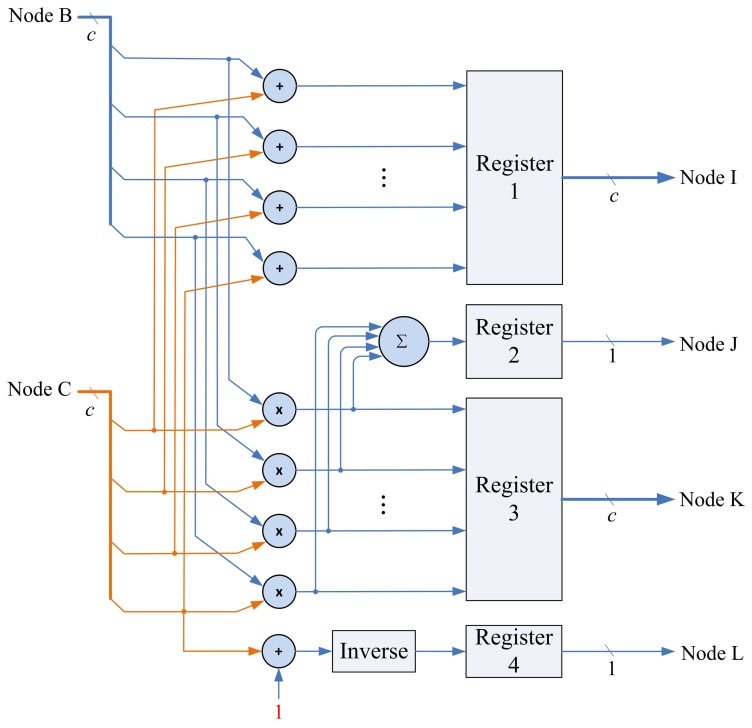
The architecture of matrix computation unit.

**Figure 12.**
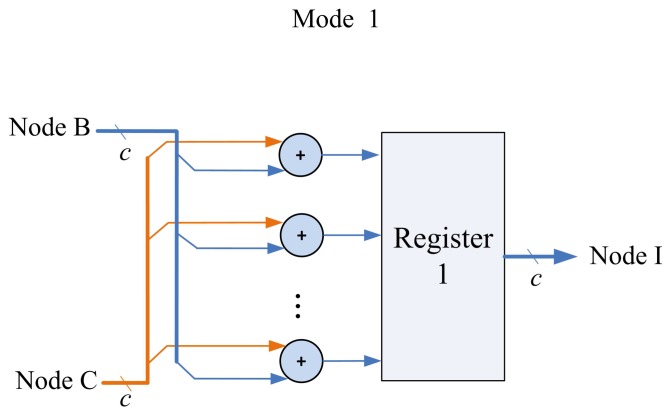

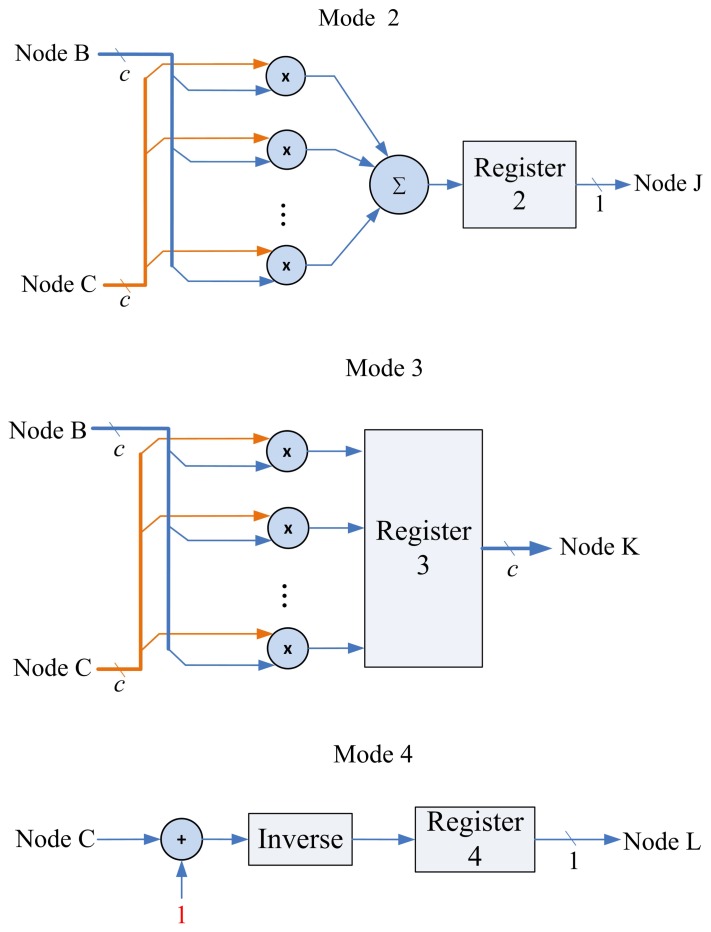
Four modes of matrix computation unit.

The matrix computation unit contains *N* 2-input multipliers, *N* 2-input adders, one *N*-input adder, and one inverse operator, as shown in Figure 11. The matrix computation unit therefore is able to perform *c* parallel multiplications and additions. The circuit operates in four modes, as shown in Figure 12. Modes 1 and 2 perform *c* parallel multiplications and additions, respectively. Mode 3 uses *c* 2-input multipliers for *c* parallel multiplications, and then uses *c*-input adder to obtain the sum of the *c* products. Mode 4 performs the inverse operation.

### 5.4. Control Unit

**Figure 13.**
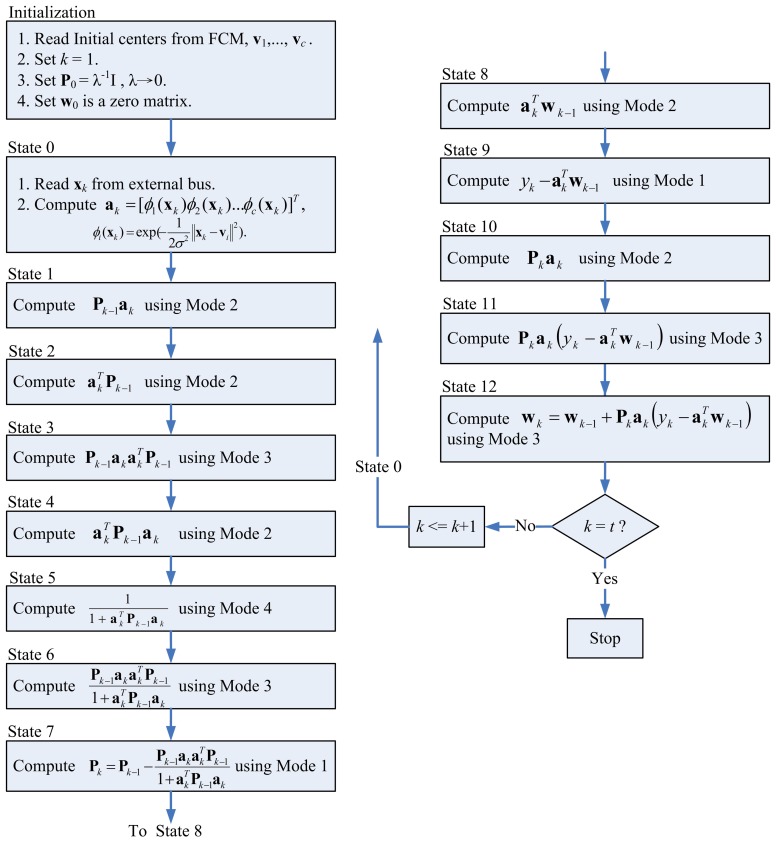
The state diagram of the control unit.

The control unit of the recursive LMS unit coordinates the operations of the kernel Gaussian computation unit, memory unit and the matrix computation unit. Figure 13 shows the state diagram of the control unit. As shown in Figure 13, the control unit operates in 13 states. State 0 reads x*_k_* from external bus, and computes a*_k_* using the kernel Gaussian Computation unit. The operations from State 1 to State 7 is to compute **P***_k_* based on Equation (18). The operations from State 8 to State 12 then finds w*_k_* based on Equation (19). All the operations from State 1 to State 12 involve the Memory unit and Matrix Computation unit. For the operation of each state, the Memory unit provides the source data. The Matrix computation unit processes the source data. The computation results are then stored back to the Memory unit.

**Figure 14.**
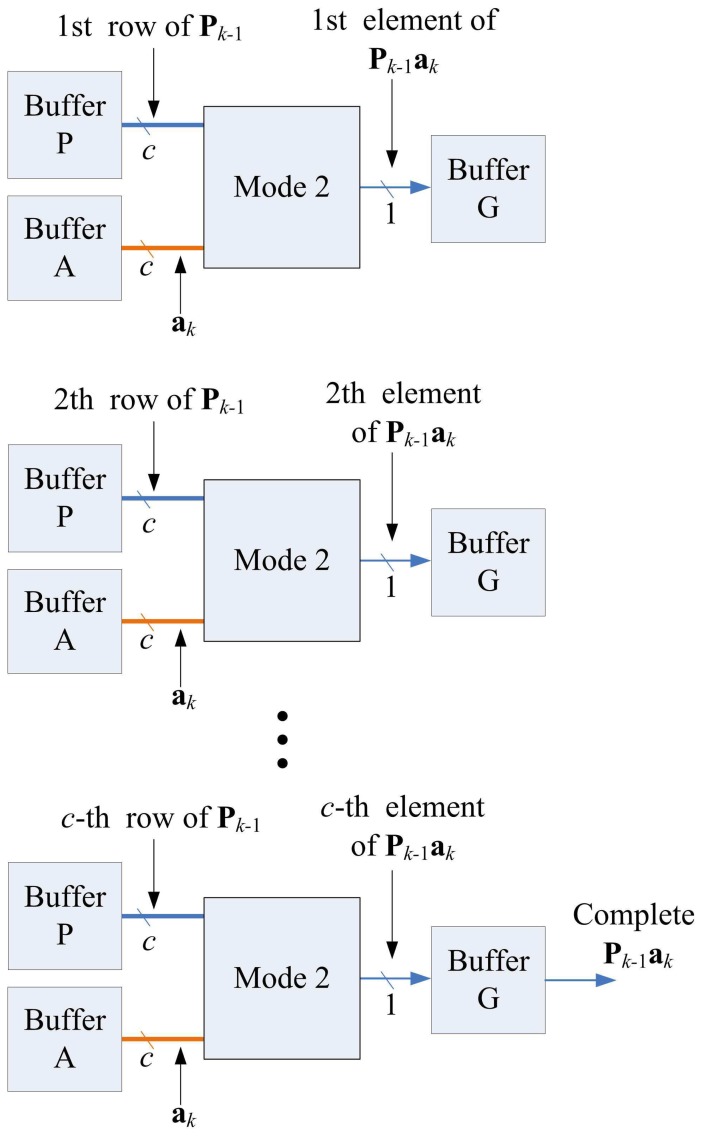
Multiple-step operations of state 1.

**Figure 15.**
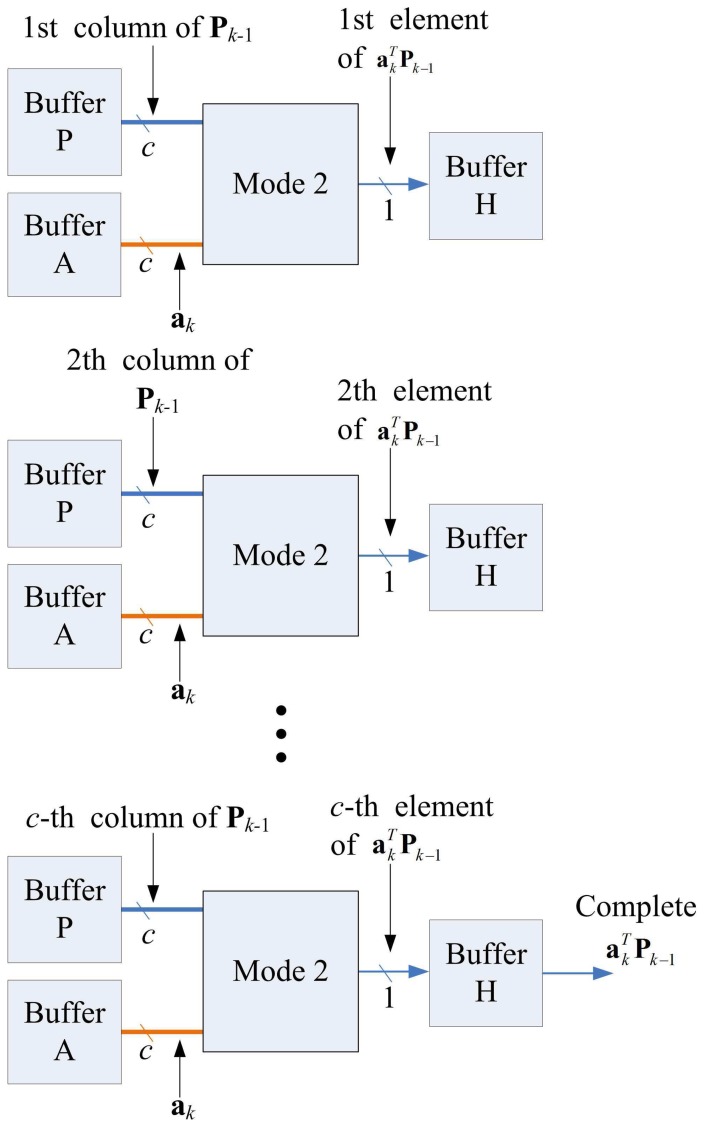
Multiple-step operations of state 2.

**Figure 16.**
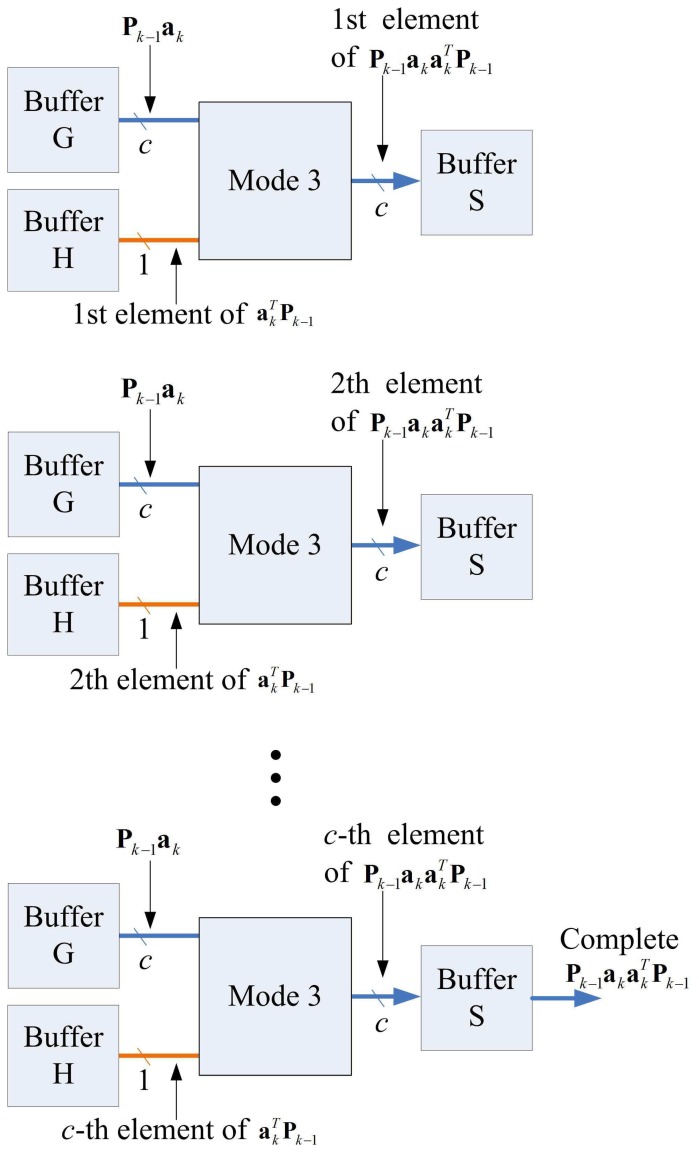
Multiple-step operations of state 3.

Note that each state may not be able to complete its operations in a single step. Because the Matrix Multiplication unit is able to perform up to *c* multiplications or additions at a time, when a state requires more than *c* multiplications or additions, multiple-step operations are required. Figure 14 shows the multiple-step operations of State 1, which compute **P***_k_*_− 1_**a***_k_*. Because there are *c*^2^ multiplications in State 1, we need *c* steps to complete the operation, as revealed in the figure. Figures 15 and 16 show the multiple-step operations of States 2 and 3, respectively. For sake of brevity, Table 2 summarizes the operations of each state. The summary consists of the source and destination buffers provided by the Memory unit, the operation mode of the Matrix Computation unit, and the number of steps required for each state.

### 5.5. The Proposed Architecture for Online RBF Training and Classification

Suppose there are *b* classes to be classified. A direct approach to use the proposed architecture for RBF training is to train the *b* classes in one shot. However, this may require large number of training vectors *t* and large number of nodes *c* in the hidden layer to achieve high classification success rate. As a result, the hardware costs of the proposed architecture may be high. An effective alternative is to train one class at a time. That is, after the training, each class has its own centers v_1_, …, v_c_ and weights w_1_, …, w_c_ for RBF classification. In addition, because each training is for a single class only, the corresponding training vectors x_1_, …, x_t_ belong to the same class. Therefore, their desired RBF output values y_1_, …, y_t_ are identical. For sake of simplicity, let y = y_1_ = … = y_t_ be the values of the desired output. During recursive LMS training, the buffer Y in the memory unit for storing desired RBF outputs should only need to be initialized as *y* before the training of each cluster. It is not necessary to update Buffer Y for each new input x_k_ during the training process.

The training process can further be simplified by allowing the desired output *y* to be identical for the training of all the clusters. In this way, the buffer Y should only be initialized before the training of the first cluster. Its value will then be reused for the training of subsequent clusters.

This simplification is also beneficial for RBF classification after the training. It is not necessary to store the desired output for individual clusters because they share the same one (i.e., *y*). Given an input vector x for RBF classification, let *y* be the output of the RBF network for class *i*, and let *E_i_* = (*ŷ* — *y*)^2^ be the squared distance between the desired output and the actual output. The vector x will be assigned to class i*, when

(26) $$i*=\arg\underset{1\leq i\leq b}{\min}E_{i}$$

The classification circuit for each class mainly contains the kernel computation unit shown in Figure 9, and c multipliers for the computation of *ŷ* based on Equation (2). It can be effectively implemented in a pipelined fashion. Replicated copies of the circuit with one for each class (i.e., *b* copies) can operate in parallel to further enhance the throughput of classification.

**Figure 17.**
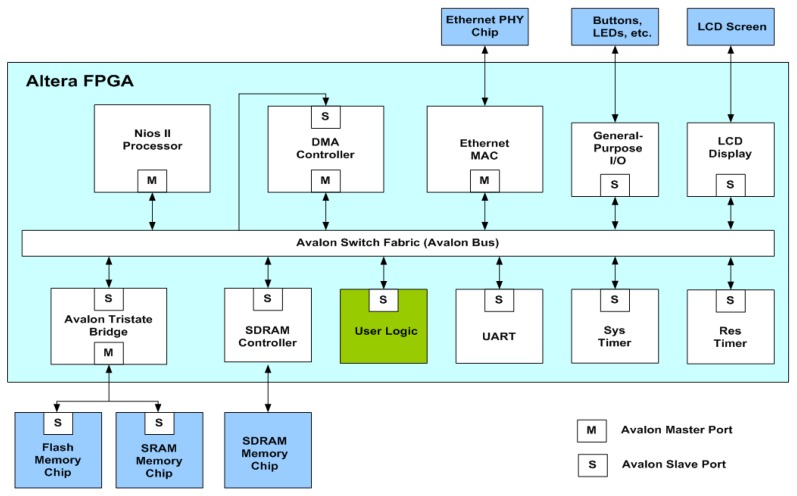
The SOPC architecture.

The proposed architecture can be employed in conjunction with the softcore processor for on-chip learning and classification. As depicted in Figure 17, the proposed architecture is used as a custom user logic in a system-on-programmable-chip (SOPC) consisting of softcore NIOS II processor, DMA controller, ethernet MAC and SDRAM controller for controlling off-chip SDRAM memory.

The NIOS II processor is used for coordinating all components in the SOPC. It receives training/test vectors from ethernet MAC and stores these vectors in the SDRAM. It is also able to deliver the training and classification results to external hosts via the ethernet MAC. In addition, the softcore processor is responsible for the initialization of the proposed architecture and DMA controller. The initialization of the proposed architecture involves the loading of the initial parameters to the FCM and recursive LMS circuits. These parameters include the number of centers *c*, the initial centers *v_i_*, *i* = 1,…, *c*, for FCM circuit, and *σ*^2^, *y*, P_0_ and w_0_ for recursive LMS circuit. The parameters are all stored in registers and can be accessed by softcore processor. Allowing these parameters to be pre-loaded by softcore processors may enhance the flexibility of the SOPC system.

The proposed architecture is only responsible for RBF training. The input vectors for the RBF training are delivered by the DMA controller. In the SOPC system, the training vectors are stored in the SDRAM. Therefore, the DMA controller delivers training vectors from the SDRAM to the proposed architecture. After the RBF training is completed, the NIOS II processor then retrieves the resulting neurons from the proposed architecture. All operations are performed on a single FPGA chip. The on-chip learning is well-suited for applications requiring both high portability and fast computation.

## 6. Experimental Results

This section presents experimental results of the proposed architecture. We first compare the proposed RBF network with existing classification techniques. The comparisons are based on datasets from the UCI database repository [21]. There are 4 datasets considered in the experiment: Balance-Scale, BCW-Integer, Iris and Wine. These datasets provide useful examples for the classification of balance scale states, breast cancer diagnosis, iris plant recognition, and wine recognition. The datasets have different sizes, number of attributes, and number of classes. The description of the datasets is shown in Table 3.

The classification success rate (CSR) is used to measure the performance of classification techniques. The CSR is defined as the number of input patterns that are successfully classified divided by the total number of input patterns. From Table 4, it can be observed that the proposed RBF network has highest CSRs for the datasets Iris and Wine. In addition, it has CSRs comparable to those of the best classifiers for the datasets Balance-Scale and BCW-Integer. The RBF network has superior performance because the centers and the connecting weights of the network can be effectively found by FCM and recursive LMS, respectively.

**Figure 18.**
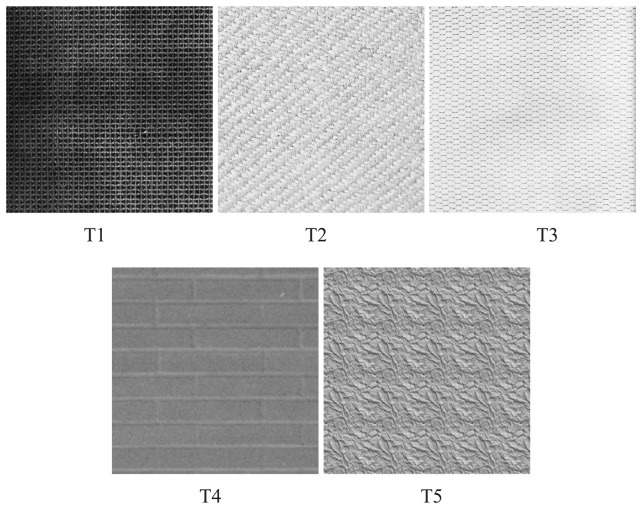
The textures considered in the experiments.

Next we compare FCM with the algorithm in [11] for selection of centers in RBF design for texture classification. The textures considered in the experiments are shown in Figure 18. The textures are labelled T1, T2, T3, T4 and T5 in the figure, respectively. The dimension of input vectors is *n* = 4 × 4. The comparisons are based on CSRs for 2-, 3- and 4-class texture classification (*i.e*., *b* = 2, 3 and 4). To achieve meaningful comparisons, all RBF networks are based on recursive LMS algorithms for the training of connecting weights. They only have different center selection algorithms. Table 5 shows the results of the comparison. For each texture classification experiment, the table reveals the largest CSR for each center selection algorithm. Because different number of centers may result in the same CSR, the lowest number of centers (*i.e*., c) yielding the CSR is also shown in the table. The RBF network with lowest *c* has the smallest hidden layer size, which is beneficial for subsequent training of connecting weights at the output layer. It can be observed from Table 5 that both center selection algorithms produce the same minimum number of centers for each experiment. For the experiment with *b* = 2, both methods also attain 100% CSR. Nevertheless, when *b* = 4, the FCM has superior CSR. Therefore, FCM is an effective alternative for RBF design.

We then evaluate the area complexities and latency of the proposed architecture. The area complexities are separated into four categories: the number of adders, the number of multipliers, the number of dividers and the number of registers. The latency is the training time. Table 6 shows the area complexities and latency of the proposed architecture. It can be observed from Table 6 that the area complexities of FCM mainly grows linearly with the vector dimension *n*. In addition, when the q-stage (*q* > 1) shift register is used, the area costs can be reduced. On the other hand, the area complexities of the recursive LMS increases with both the vector dimension *n* and the number of neurons in the hidden layer c. Moreover, the area costs grow inversely with *q*. The training time of FCM and recursive LMS increase with *c* and *q*. It also grows linearly with the number of training vectors *t*.

To further evaluate the area complexities, the physical implementation of the proposed architecture is considered. The design platform is Altera Quartus II [27] with SOPC Builder and NIOS II IDE. The target FPGA device for the hardware design is Altera Cyclone III EP3C120. Table 7 show the hardware resource consumption of the proposed architecture for vector dimensions *n* = 4 × 4 and the number of neurons *c* = 8 in the hidden layer, respectively. The FCM circuit is the basic circuit with *q* = 1. The hardware resource utilization of the entire SOPC systems is also revealed in Table 7 for comparison purpose. Three different area resources are considered in the tables: Logic Elements (LEs), embedded memory bits, and embedded multipliers. The LEs are used for the implementation of adders, dividers, and registers in the proposed architecture. Both the LEs and embedded memory bits are also used for the implementation of NIOS CPU of the SOPC system. The embedded multipliers are used for the implementation of the multipliers of the proposed architecture. It can be observed from the table that the entire SOPC consumes 84% of the LEs of the target FPGA device. Because the area costs grow with n, the extension of the circuit with *q* = 1 to larger *n* may be difficult.

Table 8 shows the effectiveness of using shift register based approach for RBF hardware design. The input vector dimension is extended from *n* = 4 × 4 to *n* = 8 × 8. That is, the vector dimension is increased 4 folds. We set *q* = 4 to reduce the number of adders, multipliers and dividers for the new vector dimension. In fact, from Table 8, we can see that the SOPC can still be implemented in the target FPGA even with *n* = 8×8. Note that, as compared with LE consumption in Table 7, the LE consumption for *n* = 8 × 8 and *q* = 4 only slightly increases from 84% to 90%. Without the employment of shift registers for vector operations, the LE consumption will exceed the capacity limit of the target FPGA device for *n* = 8 × 8.

Table 9 compares the area costs of proposed FCM with those of the FCM architecture presented in [28] for various *c* values with *q* = 1. The dimension of input vectors is *n* = 2 × 2. Because the architecture presented in [28] uses broadcasting scheme for membership coefficients and center computation, we can see from Table 9 that the proposed architecture consumes significantly less hardware resources. As the number of neurons reaches 32, the architecture presented in [28] consumes 114117 LEs, which is 97% of the LE capacity of the target FPGA device. In contrast, when *c* = 128, the proposed architecture only consumes 92295 LEs, which is 78% of the LE capacity of the target FPGA device.

The speed, area costs and CSRs of various hardware architectures for iris plant classification are shown in Table 10. The measurement of speed is the throughput, which is defined as the number of classifications per second. The hardware circuits are implemented in different FPGA devices. Therefore, it may be difficult to have direct comparisons on the hardware resource consumption. Our comparisons are based on the facts that logic cells (LCs) are the major hardware resources available in the FPGA devices for [29,30]. The LCs and LEs have similar structures, which contain a look-up table (LUT) and a 1-bit register. Therefore, the comparison of hardware resource consumption is based on the number of LEs or LCs used by the circuits. The CSR of these circuits are measured from the same dataset Iris obtained from UCI repository. There are 4 attributes for each instance. Therefore, all the circuits have the same input vector dimension *n* = 4. Two RBF networks with different number of centers (*c* = 2 and *c* = 4) are considered in this experiment. From Table 10, we can see that the proposed architectures with *c* = 2 and *c* = 4 have the highest throughput and CSR, respectively. They outperform the architecture in [29] in throughput, LE/LC consumption and CSR. They also have higher speed as compared with the architecture in [30]. The proposed RBF with *c* = 2 has slightly lower CSR as compared with the RBF with *c* = 4. However, it has higher throughput and lower LE consumption. Therefore, when both the speed and hardware consumption are important concerns, the RBF with *c* = 2 is more effective. Alternatively, when high speed computation and high CSR are desired, the RBF with *c* = 4 may be a better selection.

The proposed RBF architecture also features fast training time for texture classification. To illustrate this fact, Table 11 compares various hardware and software implementations for the training. The number of textures considered in this experiment is *b* = 4. The textures T1, T3, T4 and T5 shown in Figure 18 are used as the training images for the experiments. The CPU time of the proposed RBF architecture and the architecture in [31] are measured by the NIOS II 50 MHz softcore CPU in the SOPC platform. The architecture [31] is based on the generalized Hebbian algorithm (GHA) [2]. The number of principal components in the GHA is 7. Both the implementation of RBF and GHA architectures are based on the same FPGA device (*i.e*., Cyclone III). It can be observed from Table 11 that the proposed architecture achieves comparable CSR to that of GHA architecture with significantly lower computation time. In fact, the training time of the proposed architecture is only 4.32% of that of its software counterpart (126.68 ms *vs*. 2927.38 ms). As compared with the GHA algorithm based on incremental updating/training processes, its training time is only 2.42% of that of GHA architecture (126.68 ms *vs*. 5240.92 ms). All these facts demonstrate the effectiveness of the proposed architecture.

## 7. Concluding Remarks

A novel RBF training circuit capable of online FCM training and recursive LMS operations has been realized. Its FCM implementation consumes less hardware resources as compared with existing FCM designs. The hardware recursive LMS can also expedite the training at the output layer. Experimental results reveal that the proposed RBF network has superior CSR over existing classifiers for a bundle of datasets in the UCI repository. In addition, the proposed RBF architecture has superior speed performance over its software counterparts and other architectures for texture classification. In fact, the RBF network is able to attain CSR of 98% for Iris plant classification. Moreover, it has CSR of 96% for the 4-class texture classification. The training time of the RBF architecture is only 126.68 ms. By contrast, the training time of its software counterpart is 2927.38 ms. In addition, for the low cost FPGA devices such as Altera Cyclone III, only 84%, 60% and 39% of logic elements, embedded multipliers, and embedded memory bits are consumed for dimension *n* = 4×4. The proposed architecture therefore is an effective alternative for on-chip learning applications requiring low area costs, high CSR, and high speed computation.

## Figures and Tables

**Table 1.** The attributes of the buffers in the memory unit.

Buffers	Number of Input Ports			Number of Output Ports		Size of Buffer	Structure
Buffer Y		1		1		1	SISO
Buffer W		*c*		*c*		*c* × 1	PIPO
Buffer P		*c*		*c*		*C* × *C*	PIPO
Buffer G		*c*		*c*		1 × *c*	SIPO and PIPO
Buffer S		*c*		*c*		*C* × *C*	PIPO
Buffer H		1		*c*		1 × *c*	SISO and SIPO
Buffer T		1		1		1	SISO
Buffer A		I		*c*		1 × *c*	SIPO

**Table 2.** The operations of state 1 to state 12.

State	Source Buffers and Their Contents		Mode of Matrix Computation Unit	Destination Buffer and Its Contents	Numbers of Steps
State 1	Buffer P **P***_k_*_−1_	Buffer A **a***_k_*	Mode 2	Buffer G **P***_k_*_−1_a*_k_*	*c*
State 2	Buffer P **P***_k_*_−1_	Buffer A **a***_k_*	Mode 2	Buffer H $a_{k}^{T}P_{k-1}$	*c*
State 3	Buffer G **P***_k_*_−1_ a*_k_*	Buffer H $a_{k}^{T}P_{k-1}$	Mode 3	Buffer S $P_{k-1}a_{k}a_{k}^{T}P_{k-1}$	*c*
State 4	Buffer H $a_{k}^{T}P_{k-1}$	Buffer A **a***_k_*	Mode 2	Buffer T $a_{k}^{T}P_{k-1}a_{k}$	1
State 5	Buffer T $a_{k}^{T}P_{k-1}a_{k}$		Mode 4	Buffer T $\frac{1}{{1+a}_{k}^{T}P_{k-1}a_{k}}$	1
State 6	Buffer T $\frac{1}{{1+a}_{k}^{T}P_{k-1}a_{k}}$	Buffer S $P_{k-1}a_{k}a_{k}^{T}P_{k-1}$	Mode 3	Buffer S $\frac{P_{k-1}a_{k}a_{k}^{T}P_{k-1}}{{1+a}_{k}^{T}P_{k-1}a_{k}}$	*c*
State 7	Buffer P **P***_k_*_−1_	Buffer S $\frac{P_{k-1}a_{k}a_{k}^{T}P_{k-1}}{{1+a}_{k}^{T}P_{k-1}a_{k}}$	Mode 1	Buffer P **P**_k_	*c*
State 8	Buffer A $a_{k}^{T}$	Buffer W w*_k_*_−1_	Mode 2	Buffer T $a_{k}^{T}w_{k-1}$	1
State 9	Buffer Y *y_k_*	Buffer T $a_{k}^{T}w_{k-1}$	Mode 1	Buffer T $y_{k}-a_{k}^{T}w_{k-1}$	1
State 10	Buffer P **P**_k_	Buffer A a_k_	Mode 2	Buffer G **P**_k_a_k_	*c*
State 11	Buffer G **P**_k_a_k_	Buffer T $y_{k}-a_{k}^{T}w_{k-1}$	Mode 3	Buffer G $P_{k}a_{k}\left(y_{k}-a_{k}^{T}w_{k-1}\right)$	1
State 12	Buffer W w*_k_*_−1_	Buffer G $P_{k}a_{k}\left(y_{k}-a_{k}^{T}w_{k-1}\right)$	Mode 1	Buffer W w_k_	1

**Table 3.** Description of datasets.

	Balance-Scale	BCW-Integer	Iris	Wine
Sizes (Instances)	625	699	150	178
Attributes (*n*)	4	9	4	13
Classes (b)	3	2	3	3
Class Names	L, B, R	Benign, Malignant	Setosa, Versicolor, Virginica	1,2,3

**Table 4.** The CSRs of various classifiers for datasets from the UCI database repository [21].

Data Sets	Proposed RBF Network	Kotsiantis *et al*. [22]	Webb [23]	Zheng *et al*. [24]	De Falco *et al*. [25]	Friedman *et al*. [26]
Balance-Scale	87.04%	91.19%	75.80%	69.80%	86.88%	69.70%
BCW-Integer	97.00%	96.18%	94.86%	97.00%	97.36%	95.00%
Iris	98.00%	94.87%	97.37%	94.20%	94.74%	94.40%
Wine	98.31%	98.14%	82.23%	96.00%	97.12%	94.70%

**Table 5.** The highest CSR of the RBF networks for 2-, 3- and 4-class texture training.

Texture Classification	Proposed RBF	RBF Network in [11]
2-class (T1 & T2)	100% (*c* = 4)	100% (*c* = 4)
3-class (T1 & T3 & T4)	99.23% (*c* = 8)	99.29% (*c* = 8)
4-class (T1 & T3 & T4 & T5)	96.87% (*c* = 8)	92.47% (*c* = 8)

**Table 6.** The area complexities and latency of the proposed architecture.

	FCM	Recursive LMS	Entire RBF
Adders	*O*(*n/q*)	*O*(*c* + (*n/q*))	*O*(*c* + (*n/q*))
Multipliers	*O*(*n/q*)	*O*(*c* + (*n/q*))	*O*(*c* + (*n/q*))
Dividers	*O*(*n/q*)	*O*(1)	*O*(*n/q*)
Registers	*O*(*nc*)	*O*((*n/q*) + *c*^2^)	*O*(*nc* + (*n/q*) + *c*^2^)
Latency	*O*(*qct*)	*O*(*qt* + *ct*)	*O*((*qc* + *q* + *c*)*t*)

**Table 7.** The Hardware Resource Consumption of the Proposed Architecture with *n* = 4×4, *c* = 8 and *q* = 1.

	FCM	Recursive LMS	Entire RBF	SOPC
Logic Elements	25541/119088 (21%)	57987/119088 (49%)	88413/119088 (74%)	99817/119088 (84%)
Embedded Multiplier	79/576 (14%)	200/576 (35%)	342/576 (59%)	346/576 (60%)
Embedded Memory Bits	132864/3981312 (3%)	9799/3981312 (1%)	142913/3981312 (4%)	1558177/3981312 (39%)

**Table 8.** The hardware resource consumption of the proposed architecture with *n* = 8 × 8, *c* = 8 and *q* = 4.

	FCM	Recursive LMS	Entire RBF	SOPC
Logic Elements	30661/119088 (26%)	60035/119088 (50%)	95581/119088 (80%)	106985/119088 (90%)
Embedded Multiplier	79/576 (14%)	200/576 (35%)	342/576 (59%)	346/576 (60%)
Embedded Memory Bits	165632/3981312 (4%)	9799/3981312 (1%)	175681/3981312 (4%)	1590945/3981312 (40%)

**Table 9.** Comparisons of the area costs of various FCM architectures with *n* = 2 × 2.

c	LEs of Proposed FCM Architecture	LEs of Architecture in [28]
4	16553/119088 (14%)	21084/119088 (18%)
8	18504/119088 (16%)	35423/119088 (30%)
16	22568/119088 (19%)	59868/119088 (50%)
32	30827/119088 (26%)	114117/119088 (97%)
64	47412/119088 (40%)	NA
96	69810/119088 (59%)	NA
128	92295/119088 (78%)	NA

**Table 10.** Comparisons of different hardware architectures for Iris classification.

	FPGA Devices	Throughput	LE/LC Consumption	CSR
Polat *et al*. [29]	Xilinx Spartan 3 XC3S2000	10.0 × 10^6^	39636	96.60%
Shi *et al*. [30]	Xilinx Virtex e XCV2000E	5.4 × 10^6^	3276	98.00%
Proposed RBF (*c* = 2)	Altera Cyclone III EP3C120	30.0 × 10^6^	16147	97.33%
Proposed RBF (*c* = 4)	Cyclone III EP3C120	15.0 × 10^6^	21665	98.00%

**Table 11.** Comparisons of various implementations for texture training.

	SOPC Based on Proposed RBF Architecture	SOPC Based on GHA Architecture [31]	RBF Software
CPU Time	126.68 ms	5240.92 ms	2927.38 ms
CPU	Altera 50 MHz NIOS II	Altera 50 MHz NIOS II	Intel 1.6 GHz I5
FPGA Device	Cyclone III EP3C120	Cyclone III EP3C120	
CSR	96%	98%	97%

## References

[1] Buhmann M.D. (2004). Radial Basis Functions: Theory and Implementations.

[2] Haykin S. (2009). Neural Networks and Learning Machines.

[3] Haddadniaa J., Faeza K., Ahmadib M. (2003). A fuzzy hybrid learning algorithm for radial basis function neural network with application in human face recognition. Patt. Recogn..

[4] Pedrycz W. (1998). Conditional fuzzy clustering in the design of radial basis function neural networks. IEEE Trans. Neural Netw..

[5] Esmaeili A., Mozayani N. Adjusting the Parameters of Radial Basis Function Networks Using Particle Swarm Optimization.

[6] Alexandridis A., Chondrodima E., Sarimveis H. (2013). Radial basis function network training using a nonsymmetric partition of the input space and particle swarm optimization. IEEE Trans. Neural Netw. Learn. Syst..

[7] Sarimveis H., Alexandridis A., Bafas G. (2003). A fast training algorithm for RBF networks based on subtractive clustering. Neurocomputing.

[8] Buchtala O., Hofmann A., Sick B. (2003). Fast and efficient training of RBF networks. Lect. Note. Comput. Sci..

[9] Okamoto K., Ozawa S., Abe S. A Fast Incremental Learning Algorithm of RBF Networks with Long-Term Memory.

[10] Sing J.K., Thakur S., Basu D.K., Nasipuri M., Kundu M. (2009). High-speed face recognition using self-adaptive radial basis function neural networks. Neural Comput. Appl..

[11] Dogaru R., Dogaru I. An Efficient Finite Precision RBF-M Neural Network Architecture Using Support Vectors.

[12] Cevikbas I.C., Ogrenci A.S., Dundar G., Balkir S. VLSI Implementation of GRBF (Gaussian Radial Basis Function) Networks.

[13] Gatt E., Micallef J., Chilton E. Hardware Radial Basis Functions Neural Networks for Phoneme Recognition.

[14] Yang F., Paindavoine M. (2003). Implementation of an RBF neural network on embedded systems: Real-time face tracking and identity verification. IEEE Trans. Neural Netw..

[15] Cao B., Chang L., Li H. Implementation of the RBF Neural Network on a SOPC for Maximum Power Point Tracking.

[16] Lee G.-H., Kim S.S., Jung S. Hardware Implementation of a RBF Neural Network Controller with a DSP 2812 and an FPGA for Controlling Nonlinear Systems.

[17] Brassai S.T., Bako L., Pana G., Dan S. Neural Control Based on RBF Network Implemented on FPGA.

[18] Yang Z.-G., Qian J.-L. (2010). Hardware implementation of RBF neural network on FPGA coprocessor. Commun. Comput. Inf. Sci..

[19] Altera Corporation (2011). NIOS II Processor Reference Handbook.

[20] Altera Corporation (2008). Floating Point Exponent (ALTFP_EXP) Megafunction User Guide.

[21] Welcome to the UC Irvine Machine Learning Repository!.

[22] Kotsiantis S.B., Pintelas P.E. (2005). Logitboost of Simple Bayesian Classifier. Informatica.

[23] Webb G.I. (2000). Multiboosting: A technique for combining boosting and wagging. Mach. Learn..

[24] Zheng Z., Webb G.I. (2000). Lazy learning of Bayesian Rules. Mach. Learn..

[25] De Falco I., Cioppa A.D., Tarantino E. (2007). Facing classification problems with particle swarm optimization. Appl. Soft Comput..

[26] Friedman N., Geiger D., Goldszmidt M. (1997). Bayesian network classifiers. Mach. Learn..

[27] Documentation: Quartus II Development Software..

[28] Li H.-Y., Hwang W.-J., Chang C.-Y. (2011). Efficient fuzzy C-means architecture for image segmentation. Sensors.

[29] Polat O., Yildirim T. (2010). FPGA implementation of a General Regression Neural Network: An embedded pattern classification system. Digital Signal Process..

[30] Shi M., Bermak A., Chandrasekaran S., Amira A. An Efficient FPGA Implementation of Gaussian Mixture Models-Based Classifier Using Distributed Arithmetic.

[31] Lin S.-J., Hwang W.-J., Lee W.-H. (2012). FPGA implementation of generalized hebbian algorithm for texture classification. Sensors.

